# Meteorological Register for June, 1816

**Published:** 1816-08

**Authors:** John Bailey

**Affiliations:** Surgeon, &c.


### Meteorological Register for June, 1816.

*Kept at Harwich, in Essex.*

By JOHN BAILEY, Surgeon, &c.

| Moon's Age. | | Atmospherical Pressure. Morn. | Ev. | Temp. | Wind. | Remarks. | General Statement of Diseases. |
|---|---|---|---|---|---|---|---|
| | 1 | 29 9 | 29 9 | 70 | NW | | |
| | 2 | 8½ | 8½ | 73 | NW | | |
| ☾ | 3 | 9 | 30 | 65 | W | | Intermittens |
| | 4 | 30 | 29 8 | 60 | NW | Gale of wind | |
| | 5 | 29 8 | 6 | 59 | NW | Rain | Scarlatina |
| | 6 | 7 | 8 | 47 | N | Ditto. G. of w. | |
| | 7 | 7 | 5 | 50 | NW | Rain | Pertussis |
| | 8 | 3 | 3 | 54 | NW | ditto | |
| | 9 | 2 | 3½ | 47 | E | ditto | |
| ● | 10 | 5 | 7½ | 59 | E | ditto | |
| | 11 | 8½ | 30 | 58 | S | ditto | |
| | 12 | 30 | | 64 | S | | |
| | 13 | | 29 9 | 71 | S | | |
| | 14 | 29 9½ | 9 | 57 | NE | | |
| | 15 | 9 | | 59 | N | Gale of wind | |
| | 16 | | 9½ | 60 | NE | Dull | |
| ☽ | 17 | 9½ | 9 | 70 | NW | Clear | |
| | 18 | 8½ | | 63 | SW | | |
| | 19 | 9 | 9½ | 66 | SW | | |
| | 20 | 30 | 30 | 67 | SW | | |
| | 21 | | | 67 | S | Thick fog at night | |
| | 22 | | 29 9 | 57 | SE | Dull | |
| | 23 | 29 8 | 6½ | 62 | SW | Rain. Ditto | |
| | 24 | 7 | 8 | 61 | N | | |
| ○ | 25 | 8½ | 8½ | 68 | N | | |
| | 26 | 8 | 5½ | 55 | SE | Rain | |
| | 27 | 5 | 7½ | 57 | E | ditto | |
| | 28 | 9 | 9½ | 65 | NE | | |
| | 29 | 9½ | 9 | 69 | W | | |
| | 30 | 8 | 7 | 72 | SW | | |

Scarlatina is not prevalent at present: two very smart attacks of it have been seen in two large families, but the rest of the members of those families have escaped. Dr. Willan, in that part of his work which treats of this disease, has remarked upon this occurrence, and it has come within my observation in many instances to note the fact; it is one worthy of record, because a house so infected contains the germs of this disease, which may shoot into action many months afterwards. Some time back, an elderly woman, nurse in a very large family, arrived here, after a visit, from Ipswich. She was ill, and I visited her. The complaint proved Scarlatina Anginosa. The whole family quitted the house for many weeks. She was placed in a distant apartment; and, after her recovery, every mean was used to destroy the contagion in the room. The bedding was taken to pieces, well washed and aired, the walls scraped and white-washed, and large quantities of nitrous acid vapour, agreeably to the plan proposed for the destruction of contagion, kept for more than a week in free circulation around the area of the room, every crevice being previously stopped; yet, notwithstanding all this care and precaution, the whole family, the master of it excepted, took the infection, after they had returned home about ten days. There is no reason to imagine that they caught it elsewhere, because the disease was not known to be present in any other part of the town.

